# Total Aortic Arch Replacement With the Frozen Elephant Trunk Technique: Influence of Aortic Arch Anomalies

**DOI:** 10.1093/icvts/ivag100

**Published:** 2026-04-15

**Authors:** Alessandro Leone, Silvia Snaidero, Luca Di Marco, Chiara Nocera, Davide Pacini

**Affiliations:** Division of Cardiac Surgery, Cardiac Surgery Department, IRCCS, Azienda Ospedaliero-Universitaria di Bologna, 40138 Bologna, Italy; Department of Medical and Surgical Sciences, DIMEC, Alma Mater Studiorum—University of Bologna, Bologna, 40138, Italy; Division of Cardiac Surgery, Cardiac Surgery Department, IRCCS, Azienda Ospedaliero-Universitaria di Bologna, 40138 Bologna, Italy; Division of Cardiac Surgery, Cardiac Surgery Department, IRCCS, Azienda Ospedaliero-Universitaria di Bologna, 40138 Bologna, Italy; Department of Medical and Surgical Sciences, DIMEC, Alma Mater Studiorum—University of Bologna, Bologna, 40138, Italy; Division of Cardiac Surgery, Cardiac Surgery Department, IRCCS, Azienda Ospedaliero-Universitaria di Bologna, 40138 Bologna, Italy; Division of Cardiac Surgery, Cardiac Surgery Department, IRCCS, Azienda Ospedaliero-Universitaria di Bologna, 40138 Bologna, Italy; Department of Medical and Surgical Sciences, DIMEC, Alma Mater Studiorum—University of Bologna, Bologna, 40138, Italy

**Keywords:** frozen elephant trunk, aortic arch anomalies, aortic dissection, thoracic endovascular aortic repair

## Abstract

**Objectives:**

To assess surgical challenges and outcomes associated with arch anomalies in patients undergoing frozen elephant trunk (FET) for acute and chronic dissections.

**Methods:**

From January 2007 to January 2024, 401 patients underwent FET procedure in our centre. We included 285 patients with acute type A/B, chronic type A/B, and residual dissection. Patients were divided into normal arch group (*n* = 216) and arch anomalies group (*n* = 69), including aberrant right subclavian artery, bovine trunk, arch vertebral artery, and gothic arch.

**Results:**

Overall in-hospital mortality was 15.4% (17.4% in arch anomalies vs 14.8% in arch normal). In-hospital thoracic endovascular aortic repair (TEVAR) occurred in 7.2% (*n* = 5) of patients with arch anomalies. Stent graft-induced new entry tear at follow-up was 26.1% in arch anomalies vs 17.1% in arch normal group. Long-term survival in the overall patients was not different between the 2 groups (*P* = .383). In the subgroup of patients treated for chronic aortic dissection, freedom from TEVAR was higher in those with normal arch anatomy (*P* = .026).

**Conclusions:**

Aortic arch anomalies, especially in chronic dissection, were associated with increased endovascular reintervention. The gothic arch is the most challenging configuration due to frequent stent kinking.

## Graphical abstract

**Figure:**
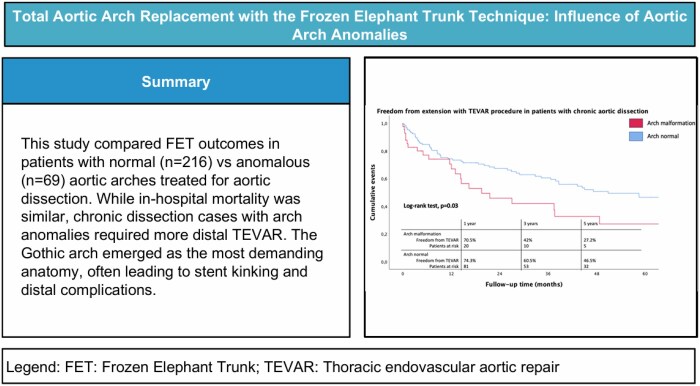

## INTRODUCTION

Frozen elephant trunk (FET) technique has become a well-established procedure for the treatment of complex aortic pathologies, particularly when secondary distal repair may be required.^1^ An increasing number of centres adopt the FET for acute aortic dissection (AAD) and chronic dissections.^2^

**Figure 1.**
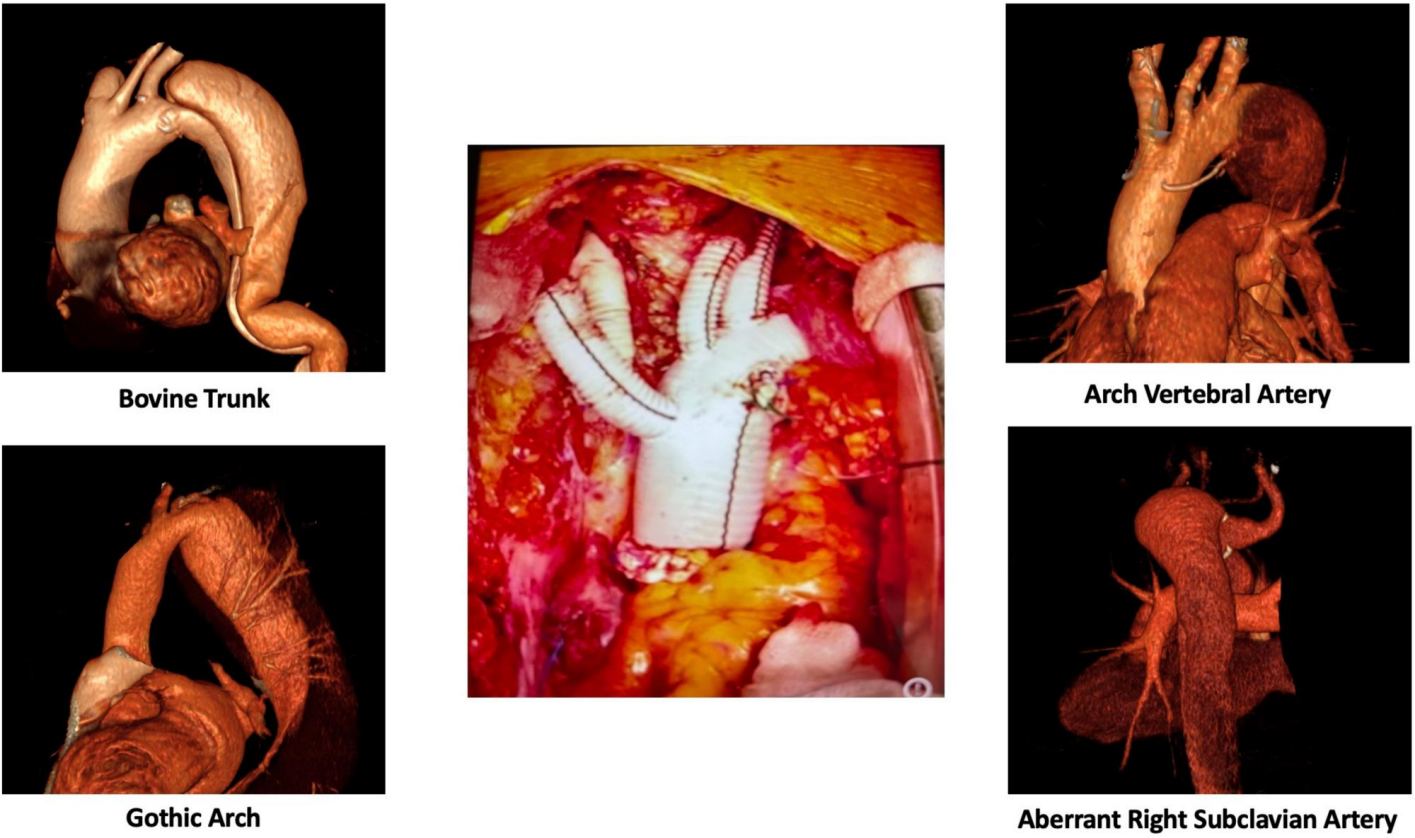
Aortic Arch Anomalies Included in Our Cohort of Patients Treated With FET Procedure for Aortic Dissection

However, patients with AAD or chronic dissection may also present congenital aortic arch anomalies (Figure 1).

While literature provides insights into outcomes, reintervention rates, and complications, few investigations have addressed the challenges of arch anomalies. These anomalies may complicate exposure, cannulation, graft deployment, and distal sealing, particularly in dissection.

This study investigates the influence of arch anomalies on surgical strategy and outcomes in patients undergoing FET for dissection, comparing them to those with normal anatomy in terms of perioperative management, morbidity and mortality, and subsequent thoracic endovascular aortic repair (TEVAR) need.

## METHODS

### Ethic statement

Patients were identified through institutional quality registries at IRCCS Azienda Ospedaliero-Universitaria di Bologna. The collection and secondary use of clinical data for research purposes are conducted in accordance with institutional policies and comply with the principles outlined in the WMA Declaration of Taipei. The study was approved by the institutional review board (IRB No.121/2022/Disp/AUOBo), which waived the need for written informed consent.

### Study design

This retrospective, observational, single-centre study was conducted at the University of Bologna. Between January 2007 and January 2024, 401 patients underwent FET. Only patients treated for acute, chronic, or residual type A/B aortic dissection were included. This resulted in a study population of 285 patients. Patient selection and reasons for exclusion are summarized in **Figure S1**.

Aortic dissection was classified according to current guideline-based definitions.^3^ Acute dissection was defined as symptom onset within 14 days, subacute dissection between 15 and 90 days, and chronic dissection beyond 90 days from symptom onset. Subacute dissections were included in the chronic dissection group.

The study population was divided into 2 groups: 216 with normal arch and 69 with anomalies: aberrant right subclavian artery (ARSA), bovine trunk, vertebral artery from the arch, and gothic arch. Gothic arch was defined on preoperative computed tomography as a pointed arch with an increased height-to-width ratio (>0.8) and an acute arch angle (<120°) between the ascending and descending aorta at the level of the arch apex.^4^

Among patients with chronic dissection, a subset underwent a planned TEVAR based on preoperative CT findings. To assess the impact of these cases on study outcomes, a sensitivity analysis excluding planned TEVAR was performed.

### Statistical analysis

Data were collected from clinical reports, CT scans, and outpatient visits. Follow-up included imaging and visits at 1 and 3 months and annually thereafter.

Continuous variables were tested for normal distribution, using the Shapiro-Wilk test and variables with a normal distribution are presented as mean ± standard deviation, whereas non-normally distributed variables are reported as median(IQR). Categorical variables are reported as absolute numbers(%). Comparisons between patients with normal aortic arch anatomy and those with aortic arch anomalies were performed using the Student’s t-test and the Mann-Whitney *U*-test, as appropriate, for continuous variables and the chi-squared test or Fisher’s exact test, as appropriate, for categorical variables.

Survival at follow-up and freedom from TEVAR were assessed using Kaplan-Meier survival analysis. Survival analysis was performed including all patients, with time zero defined as the date of surgery and all-cause mortality as the event of interest. Early postoperative deaths were included to avoid survivorship bias. Freedom from TEVAR was evaluated in the subgroup of patients treated for chronic dissection. Kaplan-Meier curves were compared using the log-rank test.

To identify independent predictors of long-term all-cause mortality, a single multivariable Cox proportional hazards regression model was constructed on the study population, including in-hospital deaths. Covariates were selected based on clinical relevance and included age, sex, AAD, surgical period, reintervention status, preoperative malperfusion, postoperative malperfusion, and postoperative tracheostomy. Surgical year was included in the multivariable Cox regression model to account for potential temporal changes in surgical techniques and perioperative management. The number of variables included in the multivariable model was deemed appropriate in relation to the sample size and number of events. Results are reported as hazard ratios (HRs) with 95% confidence intervals (CIs). Only adjusted hazard ratios are reported, as the primary aim was to assess independent predictors of long-term mortality.

All *P*-values were 2-tailed, and statistical significance was defined as *P* < .05.

Follow-up analyses were performed in patients surviving beyond 30 days after surgery. Patients with a follow-up shorter than 30 days were considered lost to follow-up. Early postoperative deaths (within 30 days) were considered observed events and were not classified as lost to follow-up. Follow-up duration was calculated for each patient as the time interval between the date of surgery and either death or last available follow-up.

All patients had complete in-hospital outcome data, while follow-up analyses were performed only in patients with available follow-up.

Statistical analyses were performed using SPSS software version 26 (IBM Corp., Armonk, NY, United States).

### Indications and sizing

In patients with chronic dissection, graft sizing was based on the true lumen diameter at the intended distal landing zone. Oversizing was avoided and generally kept below 10% to minimize the risk of distal stent graft-induced new entry (dSINE) and to preserve a stable proximal landing zone. Indications for secondary TEVAR were not based on fixed diameter thresholds, but included progressive distal aortic enlargement, persistent false lumen perfusion, development of dSINE, distal aortic degeneration, or endoleaks, as identified in CT scan follow-up. In selected patients with large diameter, a staged endovascular extension was preoperatively planned.

In AAD, FET was performed when the entry tear was in the distal arch or proximal descending aorta. In complicated acute type B dissection, it was considered in younger patients when endovascular repair was unfeasible or at high risk of retrograde type A dissection. The selected stent graft diameter was matched to the true lumen dimensions, avoiding oversizing.

Indications for secondary TEVAR were applied consistently throughout the study period and included progressive distal aortic enlargement, persistent false lumen perfusion, dSINE, distal end degeneration, or endoleaks.

### Surgical technique

All procedures were performed via full median sternotomy under cardiopulmonary bypass (CPB), using antegrade myocardial protection and mild hypothermic circulatory arrest at 25°C with bilateral antegrade selective cerebral perfusion (ASCP).^5^

Arterial cannulation strategy was individualized according to the clinical presentation and intraoperative risk assessment. Femoral artery cannulation was primarily adopted in highly unstable patients, with suspected or impending aortic rupture, pericardial effusion or tamponade, or in complex redo cases, to allow rapid establishment of CPB prior to sternotomy. In stable patients, brachiocephalic trunk (BCT) or axillary artery cannulation was preferentially used to enable early ASCP. Carotid artery cannulation was selectively employed in acute setting when it represented the only non-dissected supra-aortic vessel, and in chronic cases with extremely difficult re-entry, allowing arterial clamping to establish unilateral cerebral perfusion as a protective strategy in case of rupture.

The supraortic vessels were prepared before circulatory arrest. After resection of the arch, the descending aorta was inspected using an angioscope to confirm true-lumen identification and to detect fenestrations or entry tears. The distal anastomosis was reinforced with 3 internal pledgeted sutures and an external Teflon felt strip, after which the hybrid stent graft system was deployed. Distal stent graft insertion was performed under direct visual guidance. Once the stent graft was distally anastomosed to the aortic wall, its side branch was cannulated, and the lower body perfusion and rewarming commenced. The supra-aortic vessels were reimplanted, beginning with left subclavian artery (LSA).

Since 2014, we started implanting the FET prosthesis in arch Zone 2,^6^ with LSA clamped distally, legated proximally and reimplanted onto the graft branch. Proximal anastomosis was performed usually after this step. Finally, we performed the left common carotid artery (LCCA) and BCT reimplantation on beating heart.

In case of ARSA, reconstruction involved an end-to-end anastomosis between the distal portion of the vessel and a 10 mm Dacron graft, following closure of the vessel at its proximal origin. Subsequently, the graft is either reimplanted onto the side branch of the BCT, thereby reconstructing a neo-trunk, or onto the reperfusion side branch.^7^

The anomalous vertebral artery is always reimplanted to the side branch for the LCCA, while in case of bovine trunk, the origin of the LSA and BCT is reimplanted separately.

During the earlier years of the study, the E-vita Open prosthesis was predominantly used. From 2014 shift towards Thoraflex Hybrid prosthesis occurred, driven by technical advantages, including multibranched configuration, which facilitates supra-aortic vessel reimplantation and earlier distal reperfusion.^1^^,^^6^^,^^8^

Our strategy for FET has remained consistent over the last 17 years, including bilateral ASCP and mild hypothermia employed since 2014. The core indications for FET in aortic dissection remained consistent with contemporary guideline recommendations.^3^^,^^9^

## RESULTS

Among 285 patients treated with FET for aortic dissection, 69 (24.2%) presented an aortic arch anomaly: ARSA (*n* = 14), bovine trunk (*n* = 30), vertebral artery from the arch (*n* = 16), and gothic arch (*n* = 14). Five patients had 2 concurrent aortic arch anomalies: 2 patients with bovine trunk and gothic arch, 1 patient with bovine trunk and arch vertebral artery, 1 patient with gothic arch and ARSA, and 1 patient with ARSA and arch vertebral artery (**Table 1**).

**Table 1.** Distribution of Aortic Arch Anomalies in Overall Population and in AAD and Chronic Dissection

Arch anomalies	Overall	AAD	CAD
ARSA	14	7	7
Bovine trunk	30	13	17
Arch vertebral artery	16	6	10
Gothic arch	14	1	13

Abbreviations: AAD, acute aortic dissection; CAD, chronic aortic dissection.

Preoperative characteristics did not differ between the 2 groups (**Table 2**).

**Table 2.** Preoperative and Intraoperative Characteristics in Overall, Arch Anomaly, and Normal Arch Groups

Variable	Total (*n* = 285)	Arch anomalies (*n* = 69)	Arch normal (*n* = 216)	***P*-value** ^a^
Preoperative characteristics				
Age (years)	59.4 ± 10.9	56.8 ± 12.4	60.2 ± 10.3	.044
Male	235 (82.5%)	54 (78.3%)	181 (83.8%)	.293
COPD	22 (7.7%)	8 (11.6%)	14 (6.5%)	.166
Renal failure	13 (4.6%)	4 (5.8%)	9 (4.2%)	.572
Diabetes	11 (3.9%)	0	11 (5.1%)	.056
Smoking	108 (37.9%)	25 (36.2%)	83 (38.4%)	.744
Hypertension	239 (83.9%)	53 (76.8%)	186 (86.1%)	.068
Cerebral vasculopathy	13 (4.6%)	2 (2.9%)	11 (5.1%)	.447
Marfan	24 (8.4%)	8 (11.6%)	16 (7.4%)	.276
LVEF (%)	60 (7)	62 (12)	60 (7)	.274
Reintervention	174 (61.1%)	39 (56.5%)	135 (62.5%)	.375
AAD	81 (28.4%)	25 (36.2%)	56 (25.9%)	.098
CAD	204 (71.6%)	44 (63.8%)	160 (74.1%)	.098
Preoperative malperfusion	46 (16.1%)	10 (14.5%)	37 (17.2%)	.607
Brain	17 (6%)	2 (2.9%)	15 (6.9%)	.217
Bowel	7 (2.5%)	4 (5.8%)	3 (1.4%)	.0439
Renal	26 (9.1%)	8 (11.6%)	18 (8.3%)	.413
Lower limb	8 (2.8%)	2 (2.9%)	6 (2.8%)	.958
Urgency/emergency	94 (33%)	28 (40.6%)	66 (30.6%)	.123
Intraoperative characteristics				
E-vita	116 (40.7%)	28 (40.6%)	88 (40.7%)	.981
Thoraflex	169 (59.3%)	41 (59.4%)	128 (59.3%)	.981
Associated procedures	136 (47.7%)	36 (52.2%)	100 (46.3%)	.395
MV replacement	4 (1.4%)	1 (1.4%)	3 (1.4%)	.970
AV repair	9 (3.2%)	1 (1.4%)	8 (3.7%)	.351
AV replacement	24 (8.4%)	4 (5.8%)	20 (9.3%)	.367
CABG	18 (6.3%)	3 (4.3%)	15 (6.9%)	.440
Bentall	82 (28.8%)	22 (31.9%)	60 (27.8%)	.512
David	8 (2.8%)	3 (4.3%)	5 (2.3%)	.373
Axillary cannulation	83 (29.1%)	14 (20.3%)	69 (31.9%)	.064
Femoral cannulation	49 (17.2%)	16 (23.2%)	33 (15.3%)	.129
Ascending aorta/aortic arch cannulation	36 (12.7%)	15 (21.7%)	21 (9.8%)	.009
BCT cannulation	90 (31.6%)	17 (24.6%)	73 (33.8%)	.154
Carotid cannulation	27 (9.5%)	7 (10.1%)	20 (9.3%)	.827
CPB time (mins)	215 (89)	240 (78)	212 (93)	.433
Myocardial ischaemia time (mins)	138 (88)	180 (106)	134 (83)	.942
ASCP time (mins)	91.5 (35)	95 (55)	90 (33)	.597
Visceral ischaemia time (mins)	47 (25)	50 (22)	45 (29)	.922

a Derived from univariate analysis (Chi-square test or Fisher test for categorical variables and t-Student test or Mann-Whitney U-test for continuous variables).

Abbreviations: AAD, acute aortic dissection; ASCP, antegrade selective cerebral perfusion; AV, aortic valve; BCT, brachiocephalic trunk; CABG, coronary artery bypass graft; CAD, chronic aortic dissection; COPD, chronic obstructive pulmonary disease; CPB, cardiopulmonary bypass; LVEF, left ventricular ejection fraction; MV, mitral valve.

Chronic dissection represented the primary surgical indication overall. However, the incidence of AAD was higher among patients with arch anomalies (36.2% vs 25.9%, *P* = .098).

Mean CPB, myocardial ischaemia, ASCP, and visceral ischaemia times were comparable between groups (**Table 2**).

Overall, in-hospital mortality was 15.4% (17.4% in arch anomalies group vs 14.8% in normal arch group, *P* = .606) (**Table 3**).

**Table 3.** Postoperative and Follow-up Outcomes in Overall, Arch Anomaly, and Normal Arch Groups

Variable	Total (*n* = 285)	Arch anomalies (*n* = 69)	Arch normal (*n* = 216)	***P*-value** ^a^
Post-operative characteristics				
ICU stay (days)	4 (3)	4 (4)	4 (3)	.774
Hospital stay (days)	15 (10.25)	16 (15)	14 (9)	.675
Central neurological complications	37 (13%)	7 (10.1%)	30 (13.9%)	.421
Stroke	18 (6.3%)	2 (2.9%)	16 (7.4%)	.180
TIA	5 (1.8%)	2 (2.9%)	3 (1.4%)	.406
Coma	14 (4.9%)	3 (4.3%)	11 (5.1%)	.803
Paraplegia	6 (2.1%)	1 (1.4%)	5 (2.3%)	.663
Paraparesis	15 (5.3%)	4 (5.8%)	11 (5.1%)	.820
Visceral malperfusion	57 (20%)	12 (17.4%)	45 (20.8%)	.534
Renal	49 (17.2%)	10 (14.5%)	39 (18.1%)	.495
Bowel	16 (5.6%)	1 (6.3%)	15 (6.9%)	.152
Dialysis	54 (18.9%)	11 (15.9%)	43 (19.9%)	.462
In-hospital TEVAR	7 (2.5%)	5 (7.2%)	2 (0.9%)	.137
Tracheostomy	29 (10.2%)	5 (7.2%)	24 (11.1%)	.355
Low output	26 (9.1%)	10 (14.5%)	16 (7.4%)	.075
In-hospital death	44 (15.4%)	12 (17.4%)	32 (14.8%)	.606
Follow-up characteristics				
**Variable**	**Total (*n* = 235)**	**Arch anomalies (*n* = 55)**	**Arch normal (*n* = 180)**	***P*-value**
Mortality at follow-up	53 (22.6%)	6 (10.9%)	47 (26.1%)	.018
Neurological complications	8 (3.4%)	1 (1.8%)	7 (3.9%)	.685
dSINE	55 (23.4%)	18 (32.7%)	37 (20.6%)	.062
Distal end degeneration	26 (11.1%)	7 (12.7%)	19 (10.6%)	.653
TEVAR	101 (43%)	25 (45.5%)	76 (42.2%)	.672
Surgical redo	28 (11.9%)	7 (12.7%)	21 (11.7%)	.832
Follow-up time (months)	67.1 (55.9)	61.6 (53.7)	67.9 (56.7)	.527
Freedom from redo (months)	12.1 (32.3)	16.4 (29.4)	9.7 (32.4)	.501

a Derived from univariate analysis (Chi-square test or Fisher test for categorical variables and t-Student test or Mann-Whitney U-test for continuous variables).

Abbreviations: dSINE, distal stent graft-induced new entry; ICU, intensive care unit; TEVAR, thoracic endovascular aortic repair; TIA, transient ischaemic attack.

In-hospital TEVAR was required in 7 patients (2.5%). Indications included stent graft kinking (*n* = 2), distal landing zone shrinkage (*n* = 1), planned staged distal extension (*n* = 2), dSINE (*n* = 1), and distal re-entry between true and false lumen (*n* = 1). In arch anomalies group in-hospital TEVAR was required in 5 patients (7.2%): 2 patients with gothic arch for kinking/coarctation of the stent, 1 patient with ARSA, and 2 patients with bovine trunk.

The median follow-up time was 67.1 (55.9) months. Among patients surviving beyond 30 days (241/285, 84.6%), follow-up data were available for 235 (97.5%) patients. Distal stent graft-induced new entry occurred in 31.6% of arch anomalies group and 20.1% of normal arch group (*P* = .07) (**Table 3**).

**Figure 2.**
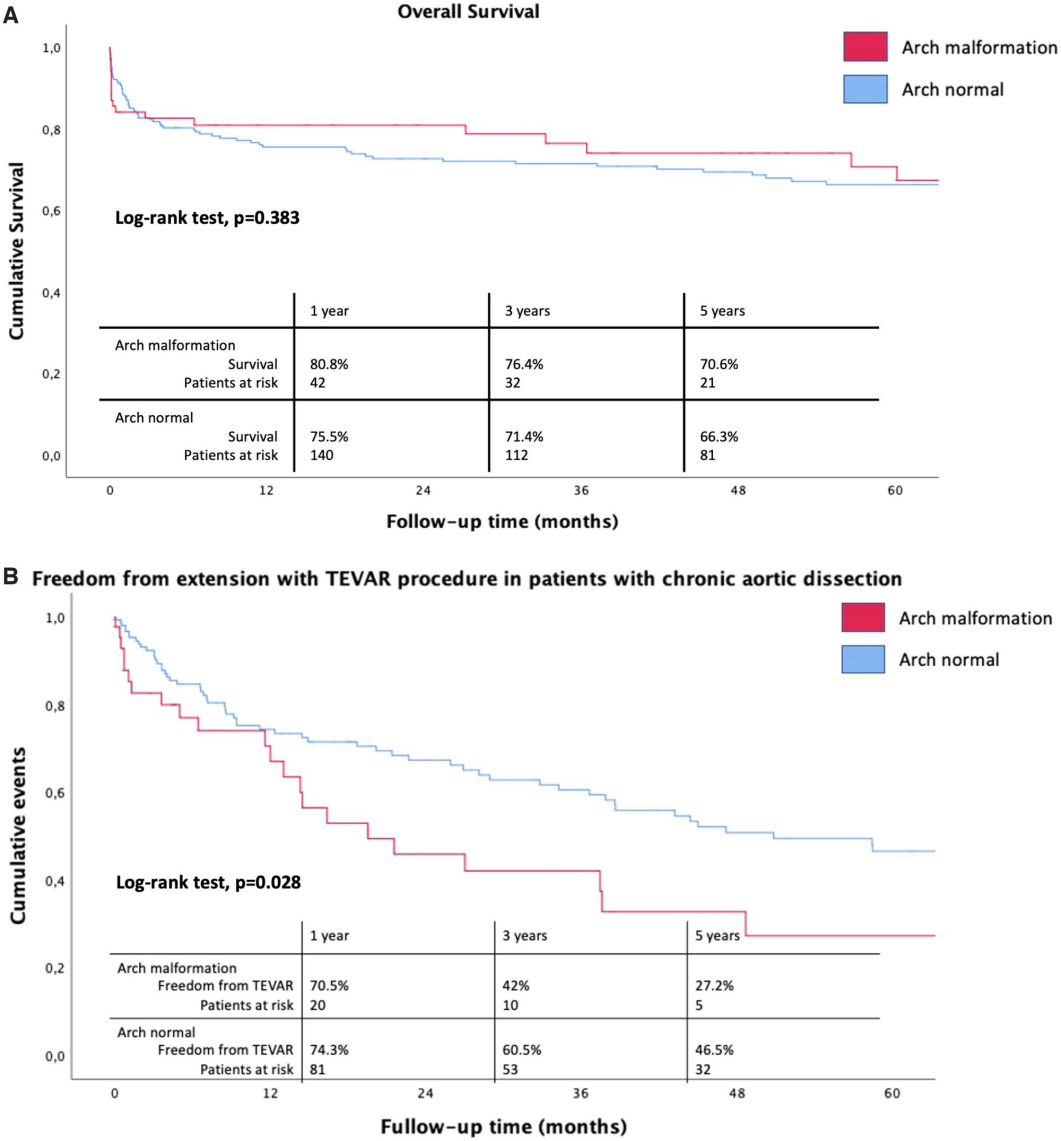
Kaplan–Meier curves showing postoperative outcomes in patients undergoing FET, comparing those with normal aortic arch versus arch anomalies, including survival and freedom from reintervention (TEVAR). (A) 5-Year cumulative survival distribution for patients with aortic dissection receiving FET procedure (normal arch vs arch anomalies). (B) 5-Year freedom from TEVAR for patients with chronic dissection receiving FET procedure (normal arch vs arch anomalies). FET: Frozen Elephant Trunk; TEVAR: Thoracic endovascular aortic repair

Survival at 1 and 5 years was 76.7% and 72.5%, respectively. Overall survival did not differ between patients with and without aortic arch anomalies at Kaplan-Meier analysis when including in-hospital and early postoperative deaths (log-rank test, *P* = .383; Figure 2**A**). Consistently, in the multivariable Cox regression model, the presence of an aortic arch malformation was not an independent predictor of mortality (HR = 0.9, 95% CI 0.6-1.6, *P* = .926) (**Table 4**).

**Table 4.** Multivariable Cox Proportional Hazards Regression Analysis for Long-Term All-Cause Mortality (Single Multivariable Adjusted Model)

Variable	HR	95% CI	***P*-value** ^a^
Arch malformation	0.9	0.6-1.6	.926
Age (years)	1.1	1.0-1.1	<.001
Gender (female)	1.4	0.8-2.3	.228
Surgical year	0.9	0.9-1.0	.626
AAD	1.8	0.9-3.6	.109
Reintervention	1.6	0.9-3.1	.117
Preoperative malperfusion	1.6	0.9-2.7	.052
Tracheostomy	1.2	0.7-2.3	.460
Postoperative malperfusion	4.6	2.9-7.3	<.001

Hazard ratios were derived from a single multivariable cox proportional hazards model including all listed covariates.

a Derived from single multivariable Cox regression analysis.

Abbreviations: AAD, acute aortic dissection; CI, confidence interval; HR, hazard ratio.

During follow-up, 27 patients with arch anomalies underwent TEVAR: 3 patients with AAD (1 with ARSA and 2 with bovine trunk) and 24 patients with chronic dissection (4 with ARSA, 8 with bovine trunk, 6 with arch vertebral artery and 6 with gothic arch). Freedom from TEVAR at follow-up was not statistically different between the 2 cohorts (*P* = .793); however, in patients with chronic dissection, it was significantly higher in arch anomalies group (*P* = .028) (Figure 2**B**).

Among patients with chronic dissection, 14 underwent a planned staged TEVAR. After exclusion of these planned procedures, the comparison between patients with and without aortic arch anomalies remained unchanged, confirming a higher need for secondary TEVAR in patients with arch anomalies. The majority of secondary TEVAR procedures were performed for dSINE, distal end degeneration, or endoleaks (**Table S1**).

## DISCUSSION

Originally conceived as a single-step approach for arch and descending aorta disease, FET demonstrated favourable false lumen thrombosis and provides a reliable landing zone for subsequent TEVAR.^3^^,^^10^ Recognizing the need to delve deeper into the heterogeneity of patients undergoing FET for aortic dissection, this analysis explores the role of aortic arch malformations, that are associated with earlier-onset dissection and increased wall fragility.^11^

The anatomical variants identified included ARSA, bovine trunk, arch vertebral artery, and gothic arch. Aberrant right subclavian artery occurs in approximately 0.5%-1.8% of the population and predisposes to early dissection.^12^ Bovine trunk presents an incidence of 7.2%-21% and correlates to higher dissection mortality (OR = 3.4; 95% CI, 1.2-9.8).^13^ Although it is often regarded as a common anatomical variant and is considered clinically benign in the general population, its presence may influence supra-aortic vessel management, cerebral perfusion strategies, and technical aspects, especially in emergency cases, and difficult re-entry. Arch vertebral artery, with an incidence of 2.4%-5.8%, increases complication risk.^14^ The gothic arch, characterized by increased systolic wave reflection, may result in elevated aortic wall stress and heightened susceptibility of dissection.^15^ Given their potential to alter local haemodynamics, aortic arch anomalies influence the onset of dissection and the technical complexity of surgery.

While prior literature includes mainly isolated case reports,^7^^,^^16^ our study provides a more comprehensive analysis by comparing 69 patients with arch anomalies with 216 controls.

The main finding of the present study is that, while perioperative outcomes and long-term survival after FET are comparable, the presence of aortic arch anomalies in patients with chronic aortic dissection (CAD) is associated with a significantly higher need for TEVAR during follow-up.

Vertebral arteries were successfully reimplanted directly, either to the LCCA or the LSA. No significant differences in postoperative neurological outcomes emerged compared to normal arch group.

Our findings support the hypothesis that with surgical planning and consistent use of ASCP, neurologic morbidity can be minimized regardless of arch configuration.

Aberrant right subclavian artery poses considerable technical challenges, due to its distal origin and tracheal proximity. In our series, an anatomical reconstruction was always pursued. Preoperative planning is essential to determine the most appropriate reconstructive strategy.^17^ This may involve reimplantation of the ARSA onto the first branch of the prosthesis, creating a neo-BCT, or onto the lateral perfusion branch,^7^ with ligation of the native vessel to prevent retrograde perfusion and reduce endoleak risk. The deployment of the FET stent graft ensures complete exclusion of the aberrant vessel’s ostium.

**Figure 3.**
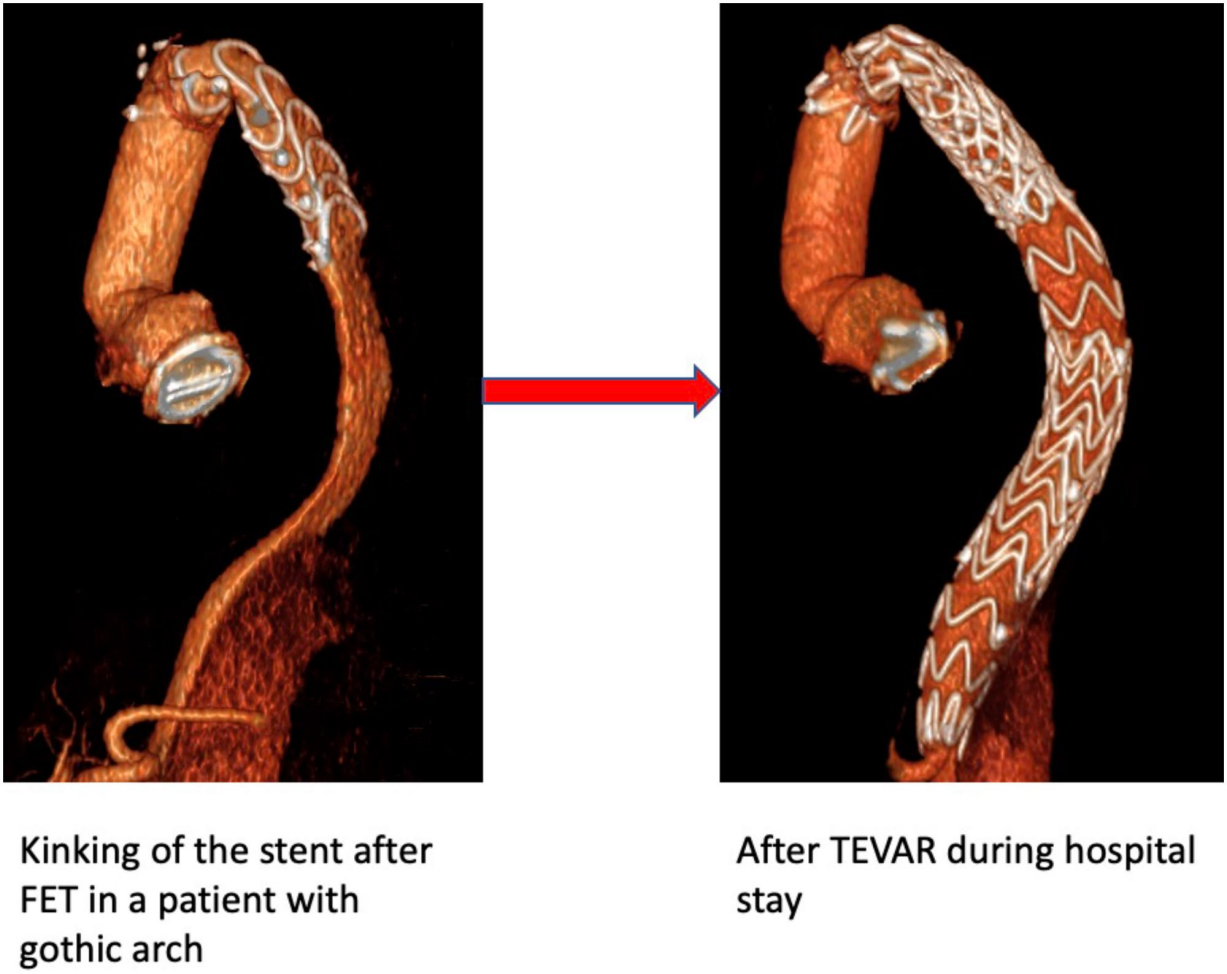
Computed tomography images showing a kinked stent graft after FET in a gothic aortic arch, with subsequent TEVAR extension to restore flow. Example of Stent Kinking After FET in a Patient with Gothic Arch, Treated with In-hospital TEVAR Extension for Visceral Malperfusion

The gothic arch posed the most significant technical challenges. These patients were more likely to develop stent graft or prosthesis kinking, often necessitating early TEVAR.^18^ Indeed, in our experience, the need for TEVAR extension during hospitalization was higher in these patients (28.5%) (Figure 3). One of the major risk factors for stent graft kinking seems to be its position at the flexure of the aorta.^19^ In our experience, the proximalization of the distal anastomosis to Zone 2 appears to be counterproductive in this setting. Indeed, the more acute curvature and shorter landing zone may predispose to mechanical complications; therefore, in patients with gothic arch, a distal anastomosis in Zone 3 may provide a more favourable angle for stent graft deployment and reduce kinking risk.

Long-term analysis showed a slightly higher incidence of TEVAR in patients with arch anomalies. When stratifying the cohort according to the primary indication for FET, a relevant disparity emerged. The incidence of TEVAR extension was higher among patients treated for chronic dissection compared to those with AAD (44.1% vs 16%, *P* < .001). Moreover, within the chronic dissection subgroup, patients with arch anomalies exhibited a significantly higher need for TEVAR than those with normal arch (*P* = .028). Importantly, the higher rate of TEVAR observed in patients with chronic dissection and arch anomalies persisted after exclusion of planned staged endovascular procedures. According to different studies,^20^^,^^21^ patients with chronic dissection are more prone to late reoperations compared to those with AAD. These findings may be explained by structural alterations of the intimo-medial layer, which become thicker, stiffer, and less responsive to remodelling.^22^ The reduced rate of false-lumen thrombosis and the progressive downstream aortic dilatation represent contributors to distal reintervention.^22^ Furthermore, dSINE is notably more frequent in chronic dissection (6%-60%) than in AAD (2.9%-15%),^23^ further predisposing to TEVAR procedures. The presence of congenital aortic arch anomalies may exacerbate these mechanisms^15^: altered flow dynamics, asymmetric branching, and intrinsic connective-tissue fragility can compromise distal sealing and remodelling. These factors explain the higher incidence of TEVAR among patients with chronic dissection and associated arch anomalies.

In addition to these results, we observed that aortic arch anomalies did not increase the perioperative and overall mortality compared with normal group. Consistently, after adjustment for potential confounders using Cox regression, the presence of an aortic arch anomaly was not independently associated with long-term mortality. The adjusted HR suggested a potentially clinically meaningful reduction in mortality risk, while the wide confidence interval indicates substantial uncertainty. Therefore, the present analysis does not allow definitive conclusions regarding the independent effect of arch anomalies on long-term survival.

Finally, although the study spans a long time period during which surgical techniques, devices, and perioperative management evolved, patients with and without aortic arch anomalies were treated throughout the entire study interval. Therefore, both groups were exposed to similar temporal trends in institutional experience and technology, limiting the risk of systematic calendar-time bias in the comparative analyses. Importantly, in the multivariable Cox regression model including surgical year as a covariate, the HR associated with aortic arch anomalies remained materially unchanged and non-significant.

### Limitation of the study

This study presented several limitations: first, it is a monocentric retrospective study, and we believe that a future multicentric prospective study may provide more definitive results. Second, the study is limited by population imbalance. Third, although the overall number of patients with aortic arch anomalies is substantial, the individual anomaly subgroups are relatively small. Another limitation is the lack of systematic preoperative quantitative assessment of false lumen patency and distal aortic diameters, which may have influenced distal aortic remodelling and the need for secondary interventions. Finally, another bias is the evolution of the technique over the last 17 years. Although adjustment for surgical year did not modify the association between arch anomalies and long-term mortality, residual confounding related to unmeasured temporal changes cannot be completely excluded. In addition, the heterogeneity of anomalies may influence the results.

## CONCLUSION

In this study, we summarized the technical challenges of performing the FET in aortic dissection, with aortic arch anomalies. Our findings underscore the difficulties posed by these anatomical variations, often associated with aortic wall fragility. Among them, the gothic arch represents the greatest challenge, both in preoperative planning and during device implantation. Therefore, preoperative assessment and evaluation for potential kinking are mandatory. This is confirmed by the higher incidence of in-hospital TEVAR and procedural complications. Although overall survival and long-term outcomes were comparable between patients with and without arch anomalies, increased requirement for TEVAR in chronic dissection emphasizes the need for preoperative planning and postoperative surveillance in this subset of patients.

## Supplementary Material

ivag100_Supplementary_Data

## Data Availability

Data supporting the findings of this study are not publicly available due to privacy or ethical restrictions. Data may be shared upon reasonable request from the corresponding author.

## ABBREVIATIONS

AAD: Acute aortic dissection
ARSA: Aberrant right subclavian artery
ASCP: Antegrade selective cerebral perfusion
AV: Aortic valve
CAD: Chronic aortic dissection
BCT: Brachiocephalic trunk
BMI: Body mass index
CABG: Coronary artery bypass graft
COPD: Chronic obstructive pulmonary disease
CPB: Cardiopulmonary bypass
FET: Frozen elephant trunk
ICU: Intensive care unit
LCCA: Left common carotid artery
LSA: Left subclavian artery
LVEF: Left ventricular ejection fraction
MV: Mitral valve
dSINE: Distal stent graft-induced new entry
TEVAR: Thoracic endovascular aortic repair
TIA: Transient ischaemic attack
