# Supplementary material for: Total Aortic Arch Replacement With the Frozen Elephant Trunk Technique: Influence of Aortic Arch Anomalies
Source: Interdiscip Cardiovasc Thorac Surg. 2026 Apr 15;41(5):ivag100. doi: 10.1093/icvts/ivag100 (PMC13152671; doi:10.1093/icvts/ivag100)
Supplement: ivag100_Supplementary_Data [file ivag100_supplementary_data.zip › Supplementary Table S1 revised copia.docx]

Supplementary Table S1. Indications for secondary TEVAR in patients with chronic aortic dissection according to aortic arch morphology.

| **Variable** | **Total patients with chronic aortic dissection (n=204)** | **Arch anomalies (n=44, 21.6%)** | **Arch normal (n=160, 78.4%)** | **p-value^1^** |
| --- | --- | --- | --- | --- |
| TEVAR for dSINE | 42 (20.6%) | 13 (29.5%) | 29 (18.1%) | 0.211 |
| TEVAR for distal end degeneration | 13 (6.4%) | 2 (4.5%) | 11 (6.9%) | 0.530 |
| TEVAR for endoleak | 16 (7.8%) | 2 (4.5%) | 14 (8.7%) | 0.350 |
| TEVAR for disease progression | 24 (11.8%) | 4 (9.1%) | 20 (12.5%) | 0.494 |
| Planned TEVAR | 14 (7.9%) | 3 (7.5%) | 11 (8%) | 0.913 |

*TEVAR=thoracic endovascular aortic repair; dSINE=distal stent graft-induced new entry*

*Data are expressed as number (%)*

*^1^Derived from univariate analysis (Chi-square test or Fisher test for categorical variables and t-Student test or Mann-Whitney U test for continuous variables)*
