# Supplementary figures and images for: Total Aortic Arch Replacement With the Frozen Elephant Trunk Technique: Influence of Aortic Arch Anomalies
Source: Interdiscip Cardiovasc Thorac Surg. 2026 Apr 15;41(5):ivag100. doi: 10.1093/icvts/ivag100 (PMC13152671; doi:10.1093/icvts/ivag100)

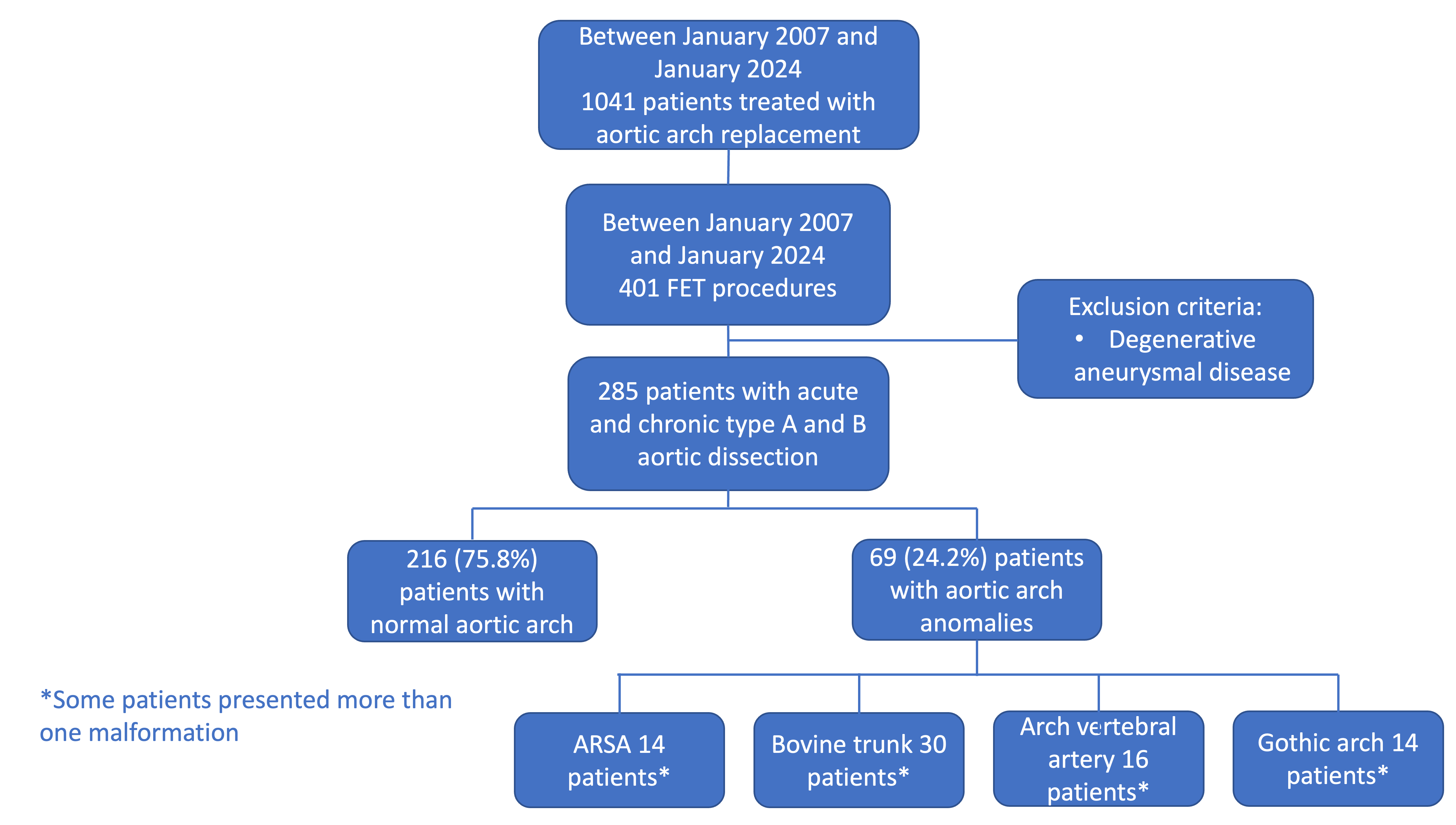

Supplement: ivag100_Supplementary_Data [file ivag100_supplementary_data.zip › Supllementary FigureS1revised.png]
